# Bilateral synchronous breast carcinomas followed by a metastasis to the gallbladder: a case report

**DOI:** 10.1186/1477-7819-5-101

**Published:** 2007-09-11

**Authors:** Flora Zagouri, Theodoros N Sergentanis, Dimitra Koulocheri, Afroditi Nonni, Aggeliki Bousiotou, Philip Domeyer, Nikolaos V Michalopoulos, Dimitrios Dardamanis, Manousos M Konstadoulakis, George C Zografos

**Affiliations:** 1 Breast Unit, 1st Department of Propaedeutic Surgery, Hippokratio Hospital, School of Medicine, University of Athens, Greece; 2 Department of Pathology, School of Medicine, University of Athens, Greece; 3 Department of Pathology, Hippokratio Hospital, Athens, Greece

## Abstract

**Background:**

Breast cancer is usually associated with metastases to lungs, bones and liver. Breast carcinoma metastasizing to the gallbladder is very rare.

**Case presentation:**

A 59-year-old woman presented with bilateral synchronous breast lesions. A palpable, retroareolar solid lesion of diameter equal to 5 cm was present in the right breast, and a newly developed, non-palpable lesion with microcalcifications (diameter equal to 0.7 cm) was present in the upper outer quadrant of the left breast. Modified radical mastectomy was performed on the right breast and lumpectomy after hook-wire localization was performed on the left breast, combined with lymph node dissection in both sides. The pathological examination revealed invasive lobular carcinoma grade II in the right breast and invasive ductal carcinoma grade I in the left breast. Chemotherapy, radiation therapy, trastuzumab and letrozole were appropriately administered. At her 18-month follow-up, the patient was free of symptoms; the imaging tests (chest CT, abdominal U/S, bone scan), biochemical tests, blood cell count and tumor markers were also normal. At the 20th month after surgery however, the patient developed symptoms of cholecystitis and underwent cholecystectomy. The histopathological examination revealed metastasis of the lobular carcinoma to the gallbladder.

**Conclusion:**

This extremely rare case confirms on a single patient the results of large series having demonstrated the preferential metastasis of lobular breast cancer to the gallbladder. Symptoms of cholecystitis should not be neglected in such patients, as they might indicate metastasis to the gallbladder.

## Background

Breast cancer is usually associated with local and lymphatic spread and with blood-borne spread to lungs, bones and liver. The central nervous system, endocrine organs, pericardium, abdominal cavity and eye are infrequently involved. Breast carcinoma metastasizing to the gallbladder is extremely rare [1], and only few cases have been reported to the literature [2-10].

The present case however is of particular interest and differentiates itself from all the previously reported cases, due to the coexistence of two independent breast carcinomas.

## Case presentation

A 59-year-old woman came to our Breast Unit due to a palpable lesion in her right breast. From the personal history, the woman had risk factors for breast cancer: positive family history for breast cancer (mother with postmenopausal breast invasive ductal carcinoma). Her BMI was equal to 24.9, and she was a housewife. The age at menarche was 12 years old, and the age at menopause was 48 years. Her reproductive history consisted of two full-term pregnancies and no spontaneous or induced abortions; the total duration of lactation was nine months. The patient reported no intake of estrogen, and her family history was negative for ovarian and prostate cancer.

Clinical examination and mammography of the right breast revealed a palpable, retroareolar lesion of diameter equal to 5 cm. In ultrasound, the consistency of the lesion was solid and multilobular. Additionally, mammography of the left breast demonstrated a newly developed, non-palpable lesion, consisting of clustered microcalcifications of diameter equal to 0.7 cm in the upper outer quadrant. Axillary lymph nodes of small size were detected on the mammogram in both sides.

FNA confirmed the malignancy on the right breast and open surgery followed. Modified radical mastectomy was performed on the right breast and lumpectomy after hook-wire localization was performed on the left breast, combined with lymph node dissection in both sides.

The pathological examination revealed:

i) in the right breast: invasive lobular carcinoma grade II, with oestrogen receptors strongly positive (3+), progesterone receptors moderately positive (2+), c-erbB-2 negative (-), and 21 out of 21 infiltrated lymph nodes.

ii) in the left breast: invasive ductal carcinoma grade I, with oestrogen receptors strongly positive (3+), progesterone receptors strongly positive (3+), c-erbB-2 (2+), and 4 out of 16 infiltrated lymph nodes.

The woman did not carry BRCA1 or BRCA2 mutations.

Chemotherapy (epirubicin (×3), CMF (×3), paclitaxel weekly) was administered; rradiation therapy followed. Subsequently, the patient took trastuzumab and then letrozole (which she is still receiving). She came regularly for the follow-up every 3 months in the first year, and at her 18-month follow-up, she was free of symptoms. The chest CT, the abdominal (upper and lower) U/S, the bone scan, the blood cell count, the biochemical tests (SGOT, SGPT, gamma-GT, ALP, potassium, sodium, ferrum, ferritin, LDH, CPK, creatinine, urea, uric acid, erythrocyte sedimentation rate) and tumor markers (CEA, CA-15-5, CA 19-9, MCA, TPA) were within normal range.

**Figure 1:**
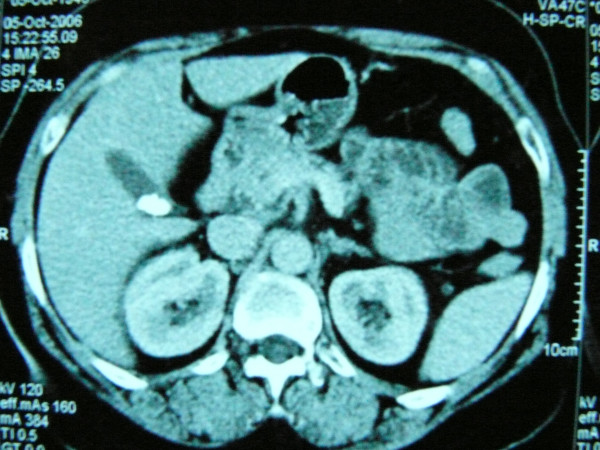
The abdominal CT of the patient, demonstrating the presence of a gallstone within the gallbladder.

At the 20th month after surgery, the patient developed symptoms of cholecystitis (a first, sudden attack of biliary pain in the right upper abdomen, which resolved within 48 hours, but was followed by mild episodes of abdominal pain within the next month). Biochemical tests were within normal range (SGOT, SGPT, gamma-GT, ALP, conjugated and unconjugated bilirubin, LDH, CPK, amylase). Ultrasound examination and CT (Figure 1) revealed the presence of gallstone disease and thus the patient underwent routine cholecystectomy. At that point, there was no indication of metastasis to the gallbladder.

**Figure 2:**
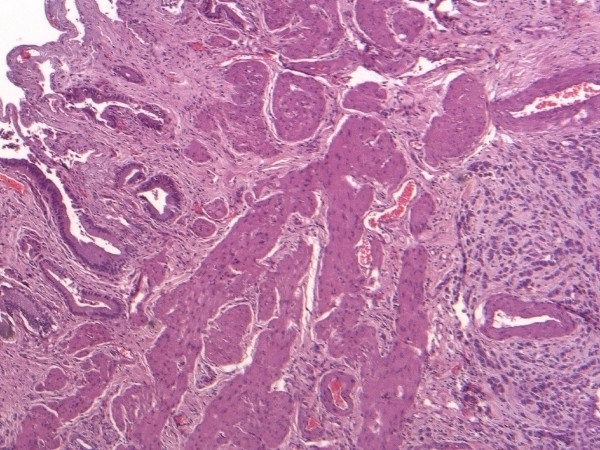
Infiltration of the gallbladder wall by the lobular breast carcinoma (H-E, ×40).

**Figure 3:**
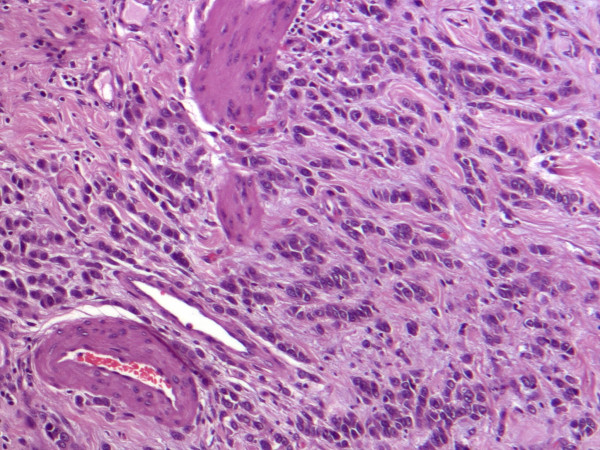
Infiltration of the gallbladder wall by the lobular breast carcinoma (H-E, ×90).

Histopathological examination, however, revealed the metastasis. At the body of the gallbladder, the wall was infiltrated (muscular layer, and adventitia) by lobular breast carcinoma (ER:3+, PR:-, cytokeratin AE1/AE3 1+) (Figures 2, 3). Additionally, the pathological evaluation showed features of chronic cholecystitis with fibrosis of the gallbladder wall.

After the establishment of the pathological diagnosis, and for the exclusion of coexisting peritoneal metastases, MRI and laparoscopy followed, which did not reveal any intra-abdominal disseminative lesions.

At the moment (32 months after the diagnosis of breast cancer, and 12 months after metastasis to the gallbladder), the patient has showed no sign of recurrence. Her clinical, laboratory, and imaging check-up is normal.

## Discussion

To our knowledge, this is the first case of two synchronous breast carcinomas (lobular invasive and ductal invasive carcinoma), one of which metastasized to the gallbladder.

Interestingly enough, the histological type that was identified in the gallbladder was lobular carcinoma. At first sight, the fact that the lobular carcinoma was accompanied by metastatic spread seems rational, given that its size was larger than that of the ductal carcinoma. However, the site of metastasis is of special importance, and the clinician may have predicted that the lobular carcinoma is the underlying cause. Indeed, lobular carcinomas show a preference to gynecologic organs, peritoneum-retroperitoneum and gastrointestinal system, including the gallbladder [11,12].

As far as the clinical presentation of metastasis to the gallbladder is concerned, symptoms of acute/chronic cholecystitis or abdominal pain prevail [2-10]. Similarly to the reported cases, symptoms of cholecystitis characterized the present case. With respect to the underlying mechanism, molecular events, such as alterations of E-cadherin expression, may be implicated in the facilitation of this rare, hematogenous metastasis [11]. Importantly, metastasis to the gallbladder has been associated with poor prognosis, while widespread metastases are frequently present at the time of diagnosis [2-10].

Of notice, there is a possibility that the clinician does not evaluate comparatively the two entities (breast cancer and gallstone disease), believing that they may share common causative factors and thus may innocently coexist; however, epidemiological studies have shown that the two diseases do not share common etiologic factors [13,14]. Taken together, the above indicate that symptoms of cholecystitis after the diagnosis and treatment of a putatively metastatic lobular breast carcinoma should not be neglected, as they might point to metastatic spread.

## Conclusion

The present case is extremely rare. Between the two synchronous breast carcinomas (lobular invasive and ductal invasive carcinoma), the lobular one metastasized to the gallbladder. This clinical observation is in line with larger series having demonstrated the preferential metastasis of lobular cancer to the gallbladder. Symptoms of cholecystitis in patient with a diagnosis of lobular carcinoma may indicate metastasis to the gallbladder.

## Competing interests

The author(s) declare that they have no competing interests.

## Authors' contributions

**FZ **conceived the idea of the study and wrote the manuscript. **TNS **interpreted the case findings with respect to international literature and wrote the manuscript. **DK **performed the radiological diagnosis and assisted at the localization of the non-palpable lesion. **AN **made the pathological diagnosis and the immunohistochemical evaluation of molecular markers in the breast specimens. **AB **made the pathological diagnosis and the immunohistochemical evaluation of molecular markers in the gallbladder specimens. **PD **performed the modified radical mastectomy and lumpectomy. **NVM **performed the cholecystectomy. **DD **performed the cholecystectomy. **MMK **performed cholecystectomy and revised critically the manuscript for important intellectual content. **GCZ **performed the modified radical mastectomy and lumpectomy, interpreted the findings, revised critically the manuscript for important intellectual content and gave the final approval of the version to be published. All authors read and approved final version of manuscript.
